# Assessing surgical trauma in robot-assisted pelvic fracture fixation: the role of the systemic immune-inflammatory index

**DOI:** 10.3389/fsurg.2025.1594928

**Published:** 2025-09-22

**Authors:** Zhang Yong-tao, Niu Jing, Chen Xin-zhi, Yang Hai-liang, He Quan-jie, Liu Huan

**Affiliations:** Department of Orthopedics, Affiliated Hospital of Hebei University of Engineering, Handan, Hebei, China

**Keywords:** robot-assisted, systemic immune-inflammation index, INFIX, anterior subcutaneous internal pelvic fixation, pelvic anterior ring fracture

## Abstract

**Objective:**

This study aims to evaluate the association between the systemic immune-inflammation index (SII) and the degree of surgical invasiveness in patients undergoing robot-assisted fixation for anterior pelvic ring fractures.

**Methods:**

This study enrolled patients aged 18–80 years with anterior pelvic ring fractures who underwent INFIX internal fixation, either with or without robotic assistance, between July 2022 and December 2023. Participants were categorized into two groups based on the use of robot-assisted techniques. Exclusion criteria included the presence of multiple fractures requiring additional internal fixation, pre-existing infections, or underlying conditions that could influence inflammatory blood markers. Operative duration, intraoperative blood loss, and incidence of lateral femoral cutaneous nerve (LFCN) injury were documented. The SII was assessed both pre- and postoperatively. Statistical analyses were performed using t-tests. Receiver operating characteristic (ROC) curves were generated to evaluate the predictive performance of SII regarding surgical invasiveness, with optimal cut-off values determined using the Youden index.

**Results:**

A total of 41 patients were included in the study. No significant differences in gender or age were observed between the robot-assisted and non-robot-assisted groups. Compared to the non-robot-assisted group, the robot-assisted group demonstrated significantly shorter operative duration, reduced intraoperative bleeding, and a lower incidence of LFCN palsy (*P* < 0.05). In addition, the postoperative SII was significantly lower in the robot-assisted group (*P* < 0.05). ROC curve analysis revealed that postoperative SII exhibited a predictive capability for surgical invasiveness, with an optimal cut-off value of 745.15 (area under the curve = 0.81; sensitivity, 75%; specificity, 83.3%).

**Conclusion:**

The findings suggest that the postoperative SII may serve as a valuable biomarker reflecting the degree of surgical invasiveness associated with robot-assisted or conventional INFIX procedures for anterior pelvic ring fractures. A postoperative SII value exceeding 745.15 demonstrates promising predictive utility for elevated surgical trauma, with a specificity of 83.3% and sensitivity of 75%.

## Introduction

Pelvic ring injuries, which are often associated with high-energy trauma, account for about 1.5%–3.9% of all fractures and are characterized by high morbidity and mortality (1). Previous studies have suggested that the posterior pelvic ring contributes 60% of the pelvis stability, compared to 40% for the anterior ring (2). Vaidya et al. introduced a subcutaneous screw rod system, now widely known as pelvic internal ﬁxator (INFIX) (3). A systematic review supports the use of INFIX for the management of unstable pelvis fractures requiring anterior ﬁxation (4). Surgical steps and ﬁxation method used varied slightly among studies, but basic steps remained the same as described by Vaidya et al. (3). In recent years, robot-assisted surgery has been introduced for the management of pelvic ring injuries, offering a novel minimally invasive approach utilizing the anterior subcutaneous INFIX technique (5–7). Some clinical indicators such as operative time, preoperative stay, length of hospitalization, blood loss, length of incision, postoperative pain scores, and blood-count-derived biomarkers were used to compare the invasiveness of various procedures in orthopedic studies (8–11). Compared to conventional open reduction internal fixation (ORIF) or free-hand screw fixation, the robotic-assisted technique offers significant advantages in managing acetabular fractures, including reduced operation time, lower intraoperative fluoroscopy frequency (IFF), fewer guide wire adjustments, less intraoperative bleeding, better screw positioning, smaller incision length, and shorter anesthesia and guide wire insertion times (12, 13).

Some blood-count-derived biomarkers such as neutrophil-to-lymphocyte ratio (NLR), the platelet-to-lymphocyte ratio (PLR), and systemic immune-inﬂammation index (SII) were employed to assess prognosis. The study by Parmana et al. (14) indicated that elevated preoperative SII values are predictors of intensive care unit stay and prolonged mechanical ventilation following off-pump coronary artery bypass graft surgery. Those biomarkers were also used in some orthopedic studies. The SII was one of the independent predictors of deep vein thrombosis (DVT) before surgery among cases developing intertrochanteric femoral fractures (15); it was one of the independent predictors of VTE after hip fracture in the elderly (16). The SII was also independently associated with increased long-term mortality in older patients with hip fractures (17). The SII exhibited a non-linear association that positively correlates with both 30-day and 1-year all-cause mortality rates (18). The PLR on postoperative day 2, and NLR and SII measurement on postoperative days 2 and 5 in patients with hip fractures were found to have significant predictive value in 1-year mortality rates (19). However, no significant correlations were observed between preoperative NLR, PLR, C-reactive protein (CRP), SII, and 3-year mortality in elderly hip fracture patients (20). In the field of vertebral fracture, the SII was positively correlated with the occurrence of vertebral compression fractures (21). According to the report in a meta-analysis, the higher SII was markedly related to poor overall survival and Enneking stage III of patients with osteosarcoma (22). Multivariate analysis revealed that age, diabetes, total albumin, SII > 752.6 × 10^9^ and a CRP > 20.25 mg/L were independent risk factors for postoperative delirium (POD), the new nomogram model can be used to accurately predict the occurrence of POD in older intertrochanteric fracture patients (23).

The application of some blood-count-derived biomarkers in the assessment of surgical trauma in orthopedics is a novel perspective. The NLR, PLR, and SII were independently related to the invasiveness of the procedures, postoperative SII was closely correlated with surgical trauma sustained by an older population with hip fractures during these surgical procedures of closed reduction internal fixation (CRIF) with a gamma nail (GN) and bipolar hemiarthroplasty (BHA) (11). However, there was no significant difference in Majeed score, healing time, and rate and postoperative complications between the robot-assisted pelvic screw fixation to the traditional fluoroscopy-assisted technique (12). As mentioned above, an elevated SII may lead to an increase in mortality rate, an increase in the incidence of VTE, the occurrence of POD, and a poor prognosis. Therefore, using SII to monitor the surgical trauma in robot-assisted surgeries may be beneficial for the assessment of prognosis.

As far as we know, there are currently no more reports on the use of SII to evaluate the surgical trauma of robot-assisted fixation of anterior pelvic ring fractures. Hence, this study aims to evaluate the correlation between SII and the level of invasiveness sustained by patients with anterior pelvic ring fracture during the robot-assisted surgical procedures.

## Methods

### Study design and patients

This single-center retrospective study analyzed data from patients with anterior pelvic ring fractures who underwent INFIX fixation between July 2022 and December 2023. Patients were allocated into two groups based on surgical technique: the robot-assisted group and the non-robot-assisted group. The study protocol was reviewed and approved by the Ethics Committee of the Affiliated Hospital of Hebei University of Engineering. The requirement for written informed consent was waived due to the retrospective nature of the study.

### Inclusion and exclusion criteria

The inclusion criteria were as follows: (1) age between 18 and 80 years; (2) a diagnosis of isolated anterior pelvic ring fracture requiring surgical fixation; (3) treatment with INFIX either with or without robotic assistance; and (4) availability of complete preoperative blood tests and a minimum follow-up of 12 weeks.

The exclusion criteria were as follows: (1) multiple concomitant fractures; (2) pre-existing conditions known to alter inflammatory or hematologic indices (e.g., hematologic malignancies, chronic renal failure, active infections, or ongoing chemotherapy/radiotherapy); (3) perioperative use of antiplatelet or anticoagulant therapy; and (4) incomplete medical records.

Patient data were collected, including gender, age, surgery duration, intraoperative bleeding, and blood routine data 1 day before surgery and 1 day after surgery. Data relating to complications such as infection, lateral femoral cutaneous nerve (LFCN) injury, and internal fixation failure were also collected. The white blood cell (WBC) count, neutrophil count, lymphocyte count, and platelet (PLT) count in blood routine should be recorded in detail. The SII was calculated using the following formula: SII = (neutrophil count × platelet count)/lymphocyte count, based on blood counts obtained 1 day after surgery. For analysis, patients were categorized into two groups: the robot-assisted group and the non-robot-assisted group.

## Surgical technique and postoperative care

### Surgical equipment and instruments

The TiRobot system, the third-generation TianJi robot for orthopedic surgery (TINAVI Medical Technologies, Beijing, China), is composed of a main console, a robotic arm, surgical planning and controlling software, an optical tracking system, a main control workstation, and a navigation and positioning toolkit. Additional surgical equipment included a C-arm x-ray machine (Siemens, Germany) and ϕ6.5 mm INFIX screw systems (BeiJing FuLe Medical Instruments, China).

#### Robot-assisted group

**Figure 1:**
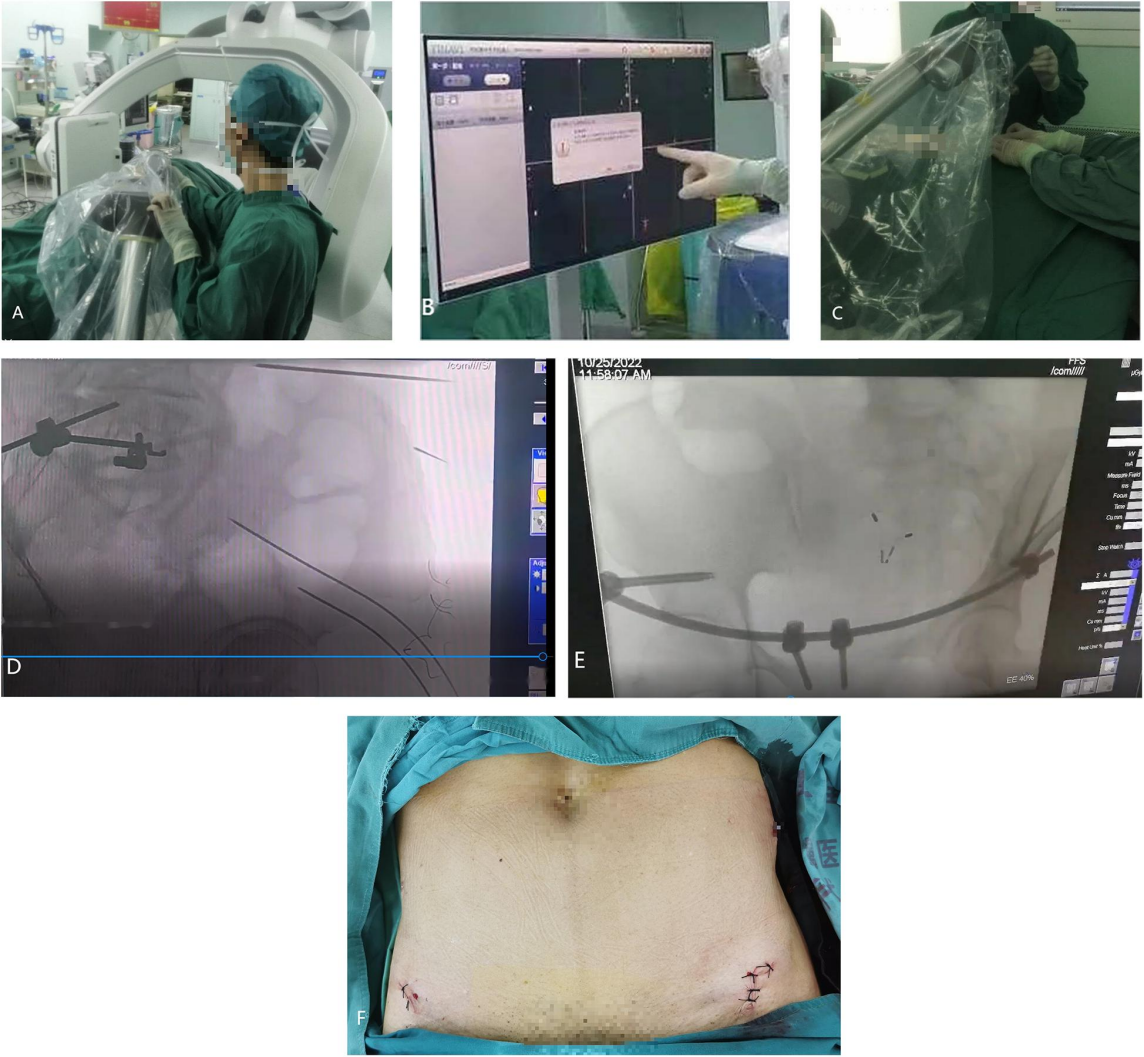
The robot-assisted surgical procedure. (**a**) Intraoperative localization 3D scan. (**b**) Transmitting image data and planning the path of the guiding needles using the robot-assisted system. (**c**) Implanting the guiding pin with the assistance of robot. (**d**) The guiding pin was implanted and was verified by a 3D scan during the operation. (**e**) The pedicle screws were implanted with the assistance of robot and verified by a 3D scan. (**f**) Surgical incision on anterior inferior iliac spine.

Patients were administered general anesthesia with tracheal intubation after being placed in the supine position. The surfaces were sterilized by routine disinfection. A tracker was fixed on the contralateral anterior superior iliac spine. Then, a sterile working environment for the robotic arm was established by assembling and fixing the robot tracker and the sterile protective sleeve. After installing the calibrator for the robot tracker, the surgeon moved to an appropriate position on the ipsilateral side near the operating bed to initiate the support systems and to lock the position of the robot. The fluoroscopic images were then transmitted to the robotic planning system. Based on the patient's anatomic features and the fracture conditions, the surgeon designed the instructions using the planning system and completed the simulation of the cannulated screw placement on the images. After a plan was established, the robotic arm began to move according to the instructions. The guidance in the pre-planned trajectory was completed outside the body using guidewires and sleeves. The skin was incised, and the subcutaneous layer was separated. The sleeve was placed onto the bone surface, and the trajectory was recalibrated. The guiding needle was inserted along the trajectory and the cannulated screws were then inserted along the needle. The positioning of the cannulated screws was verified by fluoroscopy. The specific robot-assisted process was as follows: intraoperative localization 3D mode scanning was performed and the scanned images were transmitted to a robotic system (Figures 1A,B). The guiding needles and pedicle screws were implanted with robot-assisted technology and completed with the placement of the INFIX (Figures 1C–E), and the incision was sutured (Figure 1F). The dorsal foot artery pulse should be palpated during the operation to ensure that the femoral vascular nerve is not compressed by the screw rod.

#### Non-robot-assisted group

The basic surgical steps and ﬁxation method used were same as described by Vaidya et al. (3). The C-arm fluoroscope in conventional 2D mode was used during the procedure. The guiding needles were repeated and adjusted according to the insert location and angle, and were gradually advanced under repeated optimal image intensification in two planes until the optimal anatomical location was reached. The pedicle screws were then inserted along the guiding needles, following which, INFIX was placed.

All the surgeries were performed by the same surgical team. The amount of intraoperative bleeding was calculated using the weighing method. The weight of the dry gauze was measured before the operation. The weight was measured again after the operation. The difference in weight values between the two times is the amount of intraoperative bleeding during the operation. When patients experience numbness, tingling, burning sensation, or an itchy sensation on the anterior lateral part of the thigh after surgery, these symptoms will be recorded as LFCN. When LFCN paralysis occurred, oral therapy with regular doses of mecobalamine was administered postoperative to relieve the patient's symptoms and recorded the time of recovery. Patients were given standard antibiotic prophylaxis with cefuroxime 1.5 g for 24 h after operation. Based on our single-center workflow, we conducted blood cell tests for all surgical patients on the first day after the operation. This ensures the homogeneity of the patient data. The postoperative follow-up was not less than 12 weeks.

### Statistical analysis

For the statistical analysis, SPSS version 26.0 (IBM Corp., Armonk, NY, USA) was employed. Continuous variables are presented as mean ± standard deviation (SD). Differences between groups were assessed using independent samples *t*-tests, while paired samples *t*-tests were applied for within-group comparisons. Categorical data are expressed as frequencies and percentages (%) and were compared using the chi-square (*χ*^2^) test. Measurement data were expressed as mean ± SD, and independent sample *t* test and paired sample *t* test were used. Categorical data were expressed as frequencies and percentages (%), and were compared using the *χ*^2^ test. A two-sided *P*-value of less than 0.05 was considered statistically significant. In addition, the receiver operating characteristic (ROC) curve analysis was performed to evaluate predictive performance. The optimal cut-off values were selected based on the Youden index (Youden index = sensitivity + speciﬁcity − 1, ranges from 0 to 1).

## Results

### Patient characteristics and surgical outcomes

A total of 41 patients were included in the analysis. The robot-assisted group comprised 12 males and 8 females with a mean age of 45.1 ± 14.0 years (range: 21–78), while the non-robot-assisted group included 11 males and 10 females with a mean age of 50.1 ± 11.2 years (range: 34–72). No significant differences were observed in gender distribution or age between the two groups (*P* > 0.05). INFIX implantation was successful in all patients. The robot-assisted group demonstrated a significantly shorter operative duration and reduced intraoperative blood loss compared to the non-robot-assisted group (*P* < 0.005; Table 1).

**Table 1:** Baseline characteristics in the two groups.

Group	Numbers of cases (*n*)	Gender (*n*), male/female	Age (years), mean ± SD	Surgery duration (min), mean ± SD	Intraoperative bleeding (mL), mean ± SD	LFCN injury, *n* (%)
Robot-assisted	20	12/8	45.1 ± 14.0	68.5 ± 10.0	48.4 ± 9.0	0 (0)
Non-assisted	21	11/10	50.1 ± 11.2	98.3 ± 11.7	104.3 ± 17.8	2 (9.5)
*t*-value/*χ*^2^		0.900	−1.23	−0.8665	−12.516	44.051
*P-value*		0.343	0.214	0.000	0.000	0.000

SD, standard deviation; LFCN, lateral femoral cutaneous nerve.

### Inflammatory and hematologic parameters

No significant intergroup differences were observed in preoperative WBC or PLT counts (*P* > 0.05). Postoperative WBC and PLT counts were significantly elevated compared to preoperative levels in both groups (*P* < 0.05; Table 2). Similarly, the SII increased significantly after surgery in both cohorts (*P* < 0.05; Table 3).

**Table 2:** Comparison of the differences between the two groups.

Groups	Pre-operation	Post-operation	Pre-operation	Post-operation	Pre-operation	Post-operation
	SII	SII	WBC (×10 3/µL)	WBC (×10 3/µL)	PLT (×10 3/µL)	PLT (×10 3/µL)
Robot-assisted	475.0 ± 91.7	680.3 ± 98.4	7.0 ± 0.8	8.13 ± 0.57	216.6 ± 39.9	255.1 ± 36.9
Non-assisted	379.0 ± 38.7	775.4 ± 56.6	6.8 ± 0.7	9.15 ± 0.56	209.1 ± 21.3	289.5 ± 21.1
*t*-value	4.409	−3.3815	0.748	−0.5818	0.751	−3.681
*P-*value	0.000	0.000	0.459	0.000	0.457	0.001

SII, systemic immune-inflammation index; WBC, white blood cell; PLT, platelet.

**Table 3:** Comparison of preoperative and postoperative SII differences within two groups.

Groups	SII value	Mean ± SD	*t*-value	*P -*value	95% CI
Robot-assisted	Pre-operation	475.0 ± 91.7	−31.888	0.000	−218.78 to −191.83
	Post-operation	680.3 ± 98.4			
Non-assisted	Pre-operation	379.0 ± 38.7	−47.848	0.000	−413.68 to −379.12
	Post-operation	775.4 ± 56.6			

SII, systemic immune-inflammation index.

### ROC curve analysis

**Figure 2:**
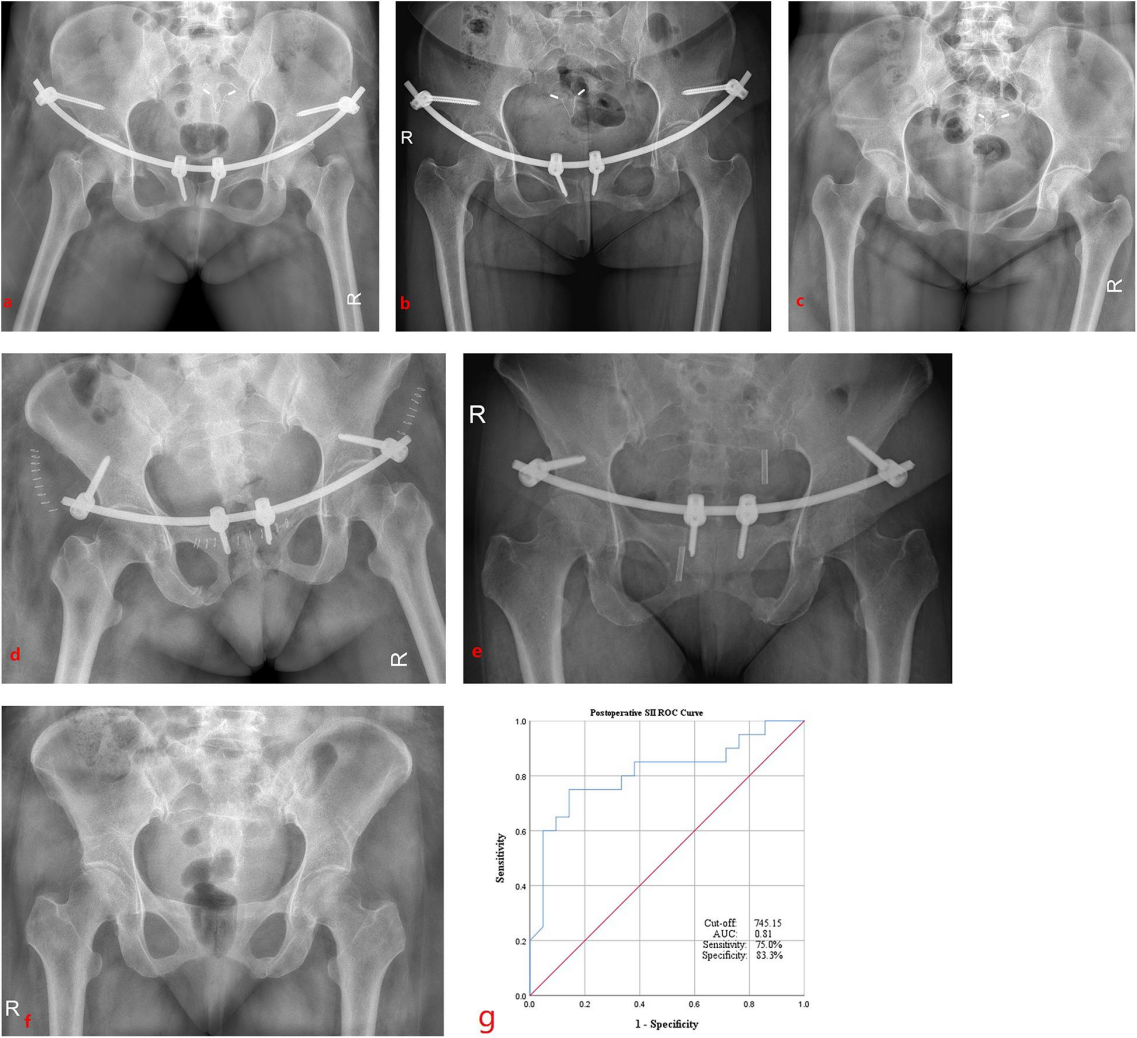
Radiographic outcomes of robot-assisted and non-assisted groups (post-op day 1, 6 months, and fixation removal) and the ROC curve analysis of postoperative SII. Robot-assisted INFIX fixation at 1 day (**a**), at 6 months (**b**), and the removal of fixation (**c**) after surgery; non-robotic-assisted INFIX fixation at 1 day (**d**), at 6 months (**e**), and at the removal of fixation (**f**) after surgery. (**g**) ROC curve indicating the cut-off values, and the AUC, sensitivity, and specificity of postoperative SII.

ROC curve analysis was performed to evaluate the predictive value of postoperative SII for surgical invasiveness. The optimal cut-off value was determined to be 745.15, with an area under the curve (AUC) of 0.81, yielding a sensitivity of 75% and a specificity of 83.3% (Figure 2G).

### Complications and follow-up

No cases of surgical site infection or internal fixation failure occurred in either group. However, two patients (9.5%) in the non-robot-assisted group developed symptoms of LFCN palsy, manifested as localized skin numbness. At the 8-week follow-up, sensory function showed significant improvement in these patients. By 1 year postoperatively, following the removal of the INFIX implant, sensory function had returned to normal in both affected individuals.

All fractures achieved healing within 3 months post-surgery, and the internal fixation was successfully removed within 1 year for all patients (Figures 2A–F). No patients were lost to follow-up, which exceeded 12 weeks for all individuals, and no data points were missing from the analysis.

## Discussion

Recent studies have utilized inflammatory systemic biomarkers, such as NLR, PLR, and SII, to predict the surgical invasiveness in orthopedic procedures. For example, one study applied these biomarkers to evaluate the trauma associated with hip fractures in older patients (11), Similarly, postoperative NLR and PLR have been employed to assess the invasiveness high-energy bicondylar tibial plateau fractures (Schatzker types V and VI) in younger patients (24). Data demonstrated that postoperative SII was a more reliable predictor of surgical trauma than NLR and PLR in the surgery of bipolar hemiarthroplasty and GN for the patients with hip fractures (11). A higher preoperative monocyte lymphocyte ratio (MLR) was found to be significantly associated with an increased 3-year postoperative mortality risk. However, no significant correlations were observed between preoperative NLR, PLR, CRP, SII, and 3-year mortality, indicating that MLR was a useful inflammatory marker for predicting 3-year mortality in elderly hip fracture patients (20). The SII was significantly lower in patients with osteomyelitis compared to those with soft tissue infections, and there was a weak correlation between erythrocyte sedimentation rate (ESR) and SII (25). The research results indicated that SII > 752.6 and CRP > 20.25 mg/L were the predictors of the occurrence of POD in the older intertrochanteric fracture patients (23). These findings highlight the potential relevance of inflammatory biomarker in postoperative management of patients with anterior pelvic ring fractures. In the present study, we compared the difference of SII between the patients in the robot-assisted group and non-robot-assisted group during the implantation of INFIX to identify any associations with the surgical invasiveness. The outcomes showed that the postoperative SII in non-robot-assisted group (775.4 ± 56.6) was higher than that in robot-assisted group (680.3 ± 98.4) (*P* < 0.05, Table 2). The results of the ROC curve analysis indicated that a postoperative SII greater than 745.15 (AUC, 0.81; sensitivity, 75%; speciﬁcity, 83.3%) (Figure 2G) could serve as a marker indicating a further increase in surgical invasiveness in anterior pelvic ring INFIX surgery. These results demonstrate that the postoperative SII has a predictive value (sensitivity, 75%; speciﬁcity, 83.3%) for assessing surgical invasiveness in robot-assisted procedure. This ﬁnding is in concordance with the research that shows postoperative SII being a more reliable predictor factor of surgical invasiveness (11).

The anterior subcutaneous internal pelvic fixator is an innovative and minimally invasive surgical method for anterior pelvic ring fractures. Most of the studies reported that patients tolerate it well with good functional results, and there were few complications if proper precautions were taken while applying the screws and rod. The systematic review has conﬁrmed that INFIX is a reliable technique for anterior pelvic injury with excellent to good functional outcomes and limited complications (4). However, the LFCN palsy remains the most common postoperative complication owing to its special anatomical relationship with the screw entry point. The meta-analysis reported that 151/598 patients (25.3%) suffered that complication, and no clear solution has been found to decrease the chance of LFCN injury (4). Similarly, another meta-analysis of 22 studies reported that the most common complications were LFCN injury in the cases of 589 patients [overall rate 28%, 95% confidence interval (CI) 15.1%–41%] (26). A robot-assisted approach to precisely planning the path of screw implantation may reduce the chance of LFCN damage. In the present retrospective study—though limited by its small sample size—no cases of LFCN injury were observed in the robot-assisted group. This sensory deficit is likely caused by direct irritation or compression of the nerve during screw placement. These findings are consistent with a 2017 study that reported no LFCN injuries in a robot-assisted cohort of 24 patients, compared to 2 cases in a manual group of 21 patients (7). Moreover, that study also found that the robot-assisted group benefited from shorter operative times and reduced intraoperative blood loss compared to the manual group (7). These outcomes are in concordance with the present study. Nevertheless, a large number of clinical cases are also needed to confirm the feasibility and accuracy of the robot-assisted technology for the INFIX placement.

Infection was reported in 19 of 619 (3.1%) cases which was treated with antibiotics, local measure, and implant removal, and no cases of osteomyelitis were reported in the meta-analysis (4). No cases of infection occurred in the present study. ESR and CRP are traditional biomarkers for the diagnosis of the infection and inflammation in clinics. One study reported that ESR and CRP do not seem to be sensitive for the diagnosis of early periprosthetic joint infections (PJI) compared with the NLR and PLR, and the postoperative NLR at the suspected time may have a great ability to predict early PJI (27). As a novel systemic inflammation indicator, SII has been identified as a more reliable predictor of surgical trauma than NLR and PLR (11). In the present study, the ROC curve analysis demonstrated that a postoperative SII > 745.15 (AUC = 0.81, 95% CI, 0.671–0.950) could serve as a predictor of increased surgical trauma in terms of speciﬁcity (83.3%) and sensitivity (75%) in the robot-assisted INFIX procedures.

The initial evidence in our study emphasize the predictive value of SII. It appears to be a sensitive indicator of systemic inflammatory states, even in the absence of postoperative infection. The clinical utility of SII is further enhanced by its economic advantages. The SII is an inexpensive, easy-to-obtain marker; it was measured from the current blood analyses performed when the patient is hospitalized and postoperatively as a basic investigation. Therefore, it does not incur any additional costs. In orthopedics, both SII and PLR had demonstrated the predict values for the diagnosis of osteoporosis in male population, which would help clinicians rapidly and conveniently diagnose osteoporosis (28). Furthermore, SII may assist clinicians in the early assessment of the probability of DVT occurrence in tibial plateau fracture patients (29). According to the existing research findings, SII was one of the independent predictors of DVT before and after surgery in hip fractures (15, 16). It has also been a predictor of increased long-term mortality in older patients with hip fractures (17). Consequently, by opting for less invasive surgical techniques (e.g., robot-assisted procedures), orthopedic surgeons may potentially mitigate the postoperative SII elevation, thereby potentially improving long-term patient outcomes. The preliminary findings from this study may inform the treatment strategy for anterior pelvic ring fractures. Patients may benefit from the robot-assisted surgical treatment. As a novel systemic inflammation indicator, SII may serve as valuable an important complement to traditional methods for assessing surgical invasiveness.

The primary limitation of this study is its single-center, retrospective design with a small sample size. Future research would benefit from a prospective, multicenter design that can enroll a larger cohort and incorporate a broader panel of inflammatory biomarkers. Second, although blood routine examinations were routinely performed, the study lacked standardized protocols for the concurrent analysis of established inflammatory markers such as CRP and ESR. Finally, postoperative blood tests were not consistently repeated at the time of discharge for all patients. Serial measurements at discharge and during subsequent follow-up visits could have provided valuable dynamic data on the temporal evolution of these biomarkers.

## Conclusion

This study demonstrated a significant correlation between the SII and surgical invasiveness in patients undergoing robot-assisted INFIX procedures. A postoperative SII value greater than 745.15 was identified as a predictive marker of increased surgical trauma, with a specificity of 83.3% and sensitivity of 75%. These findings suggest that SII may serve as a valuable, cost-effective biomarker for objectively assessing procedural invasiveness and guiding postoperative management in orthopedic trauma. However, further large-scale, prospective studies are warranted to validate its clinical utility and establish standardized application protocols.

## Data Availability

The original contributions presented in the study are included in the article/Supplementary Material, further inquiries can be directed to the corresponding author.
